# Assessment of Phytomass and Carbon Stock in the Ecosystems of the Central Forest Steppe of the East European Plain: Integrated Approach of Terrestrial Environmental Monitoring and Remote Sensing with Unmanned Aerial Vehicles

**DOI:** 10.3390/life14050632

**Published:** 2024-05-15

**Authors:** Vasiliy Slavskiy, Sergey Matveev, Sergey Sheshnitsan, Daria Litovchenko, Maxim Viktorovich Larionov, Anton Shokurov, Pavel Litovchenko, Nikolay Durmanov

**Affiliations:** 1 Faculty of Forestry, Voronezh State University of Forestry and Technologies named after G.F. Morozov, 8 Timiryazev Street, 394087 Voronezh, Russia; lisovod@bk.ru (S.M.); sheshnitsan@gmail.com (S.S.); timashchuk90@mail.ru (D.L.); pawellpv@yandex.ru (P.L.); 2 Department of Bioecology and Biological Safety, Institute of Veterinary Medicine, Veterinary and Sanitary Expertise and Agricultural Safety, Federal State Budgetary Educational Institution of Higher Education Russian Biotechnological University (ROSBIOTEC’H University), 1 Volokolamsk Highway, 125080 Moscow, Russia; 3 Computational Methods Laboratory, Mechanics and Mathematics Faculty, Lomonosov Moscow State University, Leninskiye Gory 1, Main Building, GSP-1, 119991 Moscow, Russia; shokurov.anton.v@yandex.ru; 4 Interdisciplinary Scientific and Educational School of Moscow University “Brain, Cognitive Systems, Artificial Intelligence”, Leninskiye Gory 1, Main Building, GSP-1, 119991 Moscow, Russia; 5 Ministry of Science and Higher Education of the Russian Federation, 11 Tverskaya Street, 21 Bryusov Lane, 125009 Moscow, Russia; nikolaydurmanov@gmail.com

**Keywords:** forest phytocoenoses, forest landscapes, canopy layer, morphometric parameters of trees, forest survey, phytomass, carbon stock, terrestrial environmental monitoring, remote sensing, vegetation indices, state of vegetation, landscape bioindication, synergy of methodological methods and approaches, machine/deep learning

## Abstract

The rapid and accurate estimation of aboveground forest phytomass remains a challenging research task. In general, methods for estimating phytomass fall mainly into the category of field measurements performed by ground-based methods, but approaches based on remote sensing and ecological modelling have been increasingly applied. The aim is to develop the scientific and methodological framework for the remote sensing estimation of qualitative and quantitative characteristics of forest stands, using the combination of surveys and machine learning models to determine phytomass of forest stands and calculate the carbon balance. Even-aged stands of different tree species growing in the forest steppe zone of the East European Plain were chosen as test objects. We have applied the modernized methodological approaches to compare and integrate forest and tree stand characteristics obtained by ground-based and UAV-based comprehensive surveys; additionally, we developed computer vision models and methods for determining the same characteristics by remote sensing methods. The key advantage of the proposed methodology for remote monitoring and carbon balance control over existing analogues is the minimization of the amount of groundwork and, consequently, the reduction inlabor costs without loss of information quality. Reliable data on phytomass volumes will allow for operational control of the forest carbon storage, which is essential for decision-making processes. This is important for the environmental monitoring of forests and green spaces of various economic categories. The proposed methodology is necessary for the monitoring and control of ecological–climatic and anthropogenic–technogenic transformations in various landscapes. The development is useful for organizing the management of ecosystems, environmental protection, and managing the recreational and economic resources of landscapes with natural forests and forest plantations.

## 1. Introduction

Global climate change has recently emerged as a serious long-term economic and environmental issue of universal concern [1,2]. Forest ecosystems play a significant role in the global carbon cycle; this has been confirmed by numerous scientific studies in recent decades [3,4,5,6].L. Chen et al. [7], C.I. Braga et al. [8], Y. Zhou et al. [9], A. Zielonka et al. [10], and other authors concluded that forest ecosystems contain approximately 80% of terrestrial carbon. It has been established that forest ecosystems play a significant role in reducing the concentration of greenhouse gases and mitigating global climate change [11,12,13,14,15,16,17].

Taking into account intensive forest management [18,19,20], deterioration of meteorological–climatic [21,22,23,24], and ecological [25,26,27,28,29] environmental parameters, the assessment of the bioproductivity of tree stands and forests [30,31,32,33,34] seems to be an urgent scientific and practical task. The long-term accumulation of stored carbon in forest ecosystems results in a biospheric effect related to the absorption of greenhouse gases, counteracting changes in the climate system of the planet [35,36]. Estimates of forest carbon stocks typically identify five primary pools: aboveground phytomass, belowground phytomass, deadwood (including standing and fallen dead wood), litter, and soil organic matter [3]. Aboveground phytomass is the key indicator of ecosystem productivity, forming qualitative and quantitative indicators of forest stands [37], as well as providingthe basis for the release of oxygen and carbon sinks [38]. Furthermore, determining the amount of carbon in soil and forest vegetation below ground is extremely difficult (and often impossible) when calculating plantation carbon stocks using remote sensing. The problem also lies in the obvious shortcomings of existing methods for the environmental monitoring of phytocoenoses through remote sensing.

The monitoring of the emissionand absorption of greenhouse gases by means of ground-based and remote sensing methods is extremely relevant and widely used in scientific and applied research [35]. Knowledge in the field of forest carbon accounting is currently increasing. However, different methodological approaches [38,39,40,41,42,43] and computational models [44,45,46,47,48] have been found to produce inconsistent results, highlighting the need for further methodological improvements.

This requires obtaining reliable information on the quantitative characteristics of forest stands and calculating the amounts of live and dead phytomass. Reliable estimation of phytomass in the context of climate change can provide a theoretical basis for the study of carbon cycling in terrestrial ecosystems [17,49], which has a crucial role in the response of forest ecosystems to greenhouse gas emissions [16,50]. The majority of authors [51,52,53] believe that a reliable assessment of aboveground forest phytomass will enhance the efficiency of management decisions in forest protection, conservation, and regeneration.

The most accurate method for estimating phytomass is in situ inventory with ground-based forest inventory methods [54]. The main disadvantage of the ground-based monitoring is high labor intensity, as this type of work involves a significant number of field surveys being conducted over an entire study area [55]. In addition, the problems with ground-based monitoring are related to the remoteness of the number of study sites from roads and settlements, the difficulty of moving across the study area, and often the inability to reach the forest area. This is especially true for the Central Forest Steppe zone of the East European Plain.

Rapid and accurate estimation of the aboveground forest phytomass remains a challenging research task [56,57]. In general, methods for estimating phytomass fall mainly into the category of field measurements made by ground-based methods, but remote sensing [58,59] and ecological modelling approaches have been increasingly applied [60,61].

It should be noted that remote sensing methods are of primary importance for over 70% of the Russian Federation's territory [62]. This is the optimal (and sometimes the only) method of obtaining statistically reliable and current information about forests for geographically remote and hard-to-reach areas [55].

Currently, satellite monitoring and assessment methods of forest states and structures are widespread in Russia and are used to estimate carbon balance [63,64]. With proper attribution of natural (disturbance) and anthropogenic (degradation) losses [65], the research has the potential to inform decision-making by governments across. It is well known that management and organizational decisions in relation to the rationalization of forest management, afforestation, the creation of plantings, and the environmental and economic control of plantings of different target categories are not sufficiently substantiated. This is observed in different administrative territories and geographical regions of the world. Focusing on research material on this issue is characterized by relevance and has practical benefits. Optical remote sensing images of Earth at various spatial, spectral, and temporal resolutions are freely available and are used to assess forest stands at various scales. This is reflected in the scientific works of many researchers [66,67,68,69]. Despite the obvious advantages of obtaining spatial data from satellite systems, this method of assessing forest stand phytomass has a significant disadvantage: the determination of forest tree stand characteristics is performed by analyzing the canopy of stands, but the resolution of satellite images does not provide detailed information. In addition, deadwood and understory vegetation are not taken into account. Assessments of carbon sequestration in the plantations often does not take into account the layering and structure of the forest stand. Carbon stock calculations are often very fragmented and based on approximate calculations.

Therefore, it is necessary for spatial data acquisition sources (camera type used, spatial resolution, survey age, etc.) to be enhanced. New methodologies and technologies need to be developed and implemented for the objective accounting of the carbon sink, allowing for the rapid estimation of phytomass in the forest stands, including the use of artificial intelligence (AI) [56]. Lidar technology based on the collection and analysis of data obtained using the laser scanning is widely used in the forestry sector [70]. Particularly, it can be successfully applied to improve our understanding of the three-dimensional structure of the forest environment [71]. It should be noted that there is practically lack of data on carbon sequestration in the lower layers of the forest phytocoenoses (including undergrowth and undergrowth) of the Central Forest Steppe of the East European Plain. In this regard, modern data are required on the state and productivity of forest ecosystems with differentiation according to different components of phytocoenoses.

Many researchers and scientists [72,73,74,75,76,77] have noted significant prospects for the automatic decoding of forest stands and analysis of forest ecosystems, including by laser scanning methods [78,79]. Specific vertical structuresare successfully recognized by the pattern of obtained dense point cloud and its shape using Lidars, including those based on UAVs [80]. In our study we used a combination of Lidar, RGB, and hyperspectral cameras. The effectiveness of the joint use of various types of sensors has been confirmed by many authors [81,82,83,84]. Their use corresponded to the purpose, theme, and problems of the work under consideration, as well as the idea of ensuring the synergy of advanced methodological approaches and technical means. This was necessary in accordance with the scientific idea and the specifics of our work.

Thus, the application of integrated ultra-high-resolution equipment during aerial survey [85,86] significantly increases informative and expands functional capabilities in phytomass assessment; it allows all silvicultural, bioecological, and tree stand characteristics to be identified, enabling us to determine carbon sinks in forests. Remote sensing is an extremely relevant and rapidly developing research area in the forestry industry. Remote sensing methods are widely in demand for use in assessing and monitoring forest stands.

The aim of the work is to develop the scientific and methodological basis for remote estimation of quantitative and qualitative characteristics of tree stands and ecosystems using colored light (RGB), Lidar, and hyperspectral surveys based on UAV and the presently widely accessible machine learning models (artificial intelligence)to determine aboveground phytomass of the forest stands and phytocoenoses. 

Our proposed methodological approach for the remote ecological monitoring of the productivity of aboveground phytomass of forest ecosystems based on UAVs will provide more accurate and objective information about the state and structure of phytocoenoses. This methodological approach makes it possible to determine the carbon stock in natural forest stands and plantings and to carry out an accounting of forest resources. Our approach also allows us to monitor fire, environmental, and sanitary safety in forests.

## 2. Materials and Methods

### 2.1. Study Area

**Figure 1:**
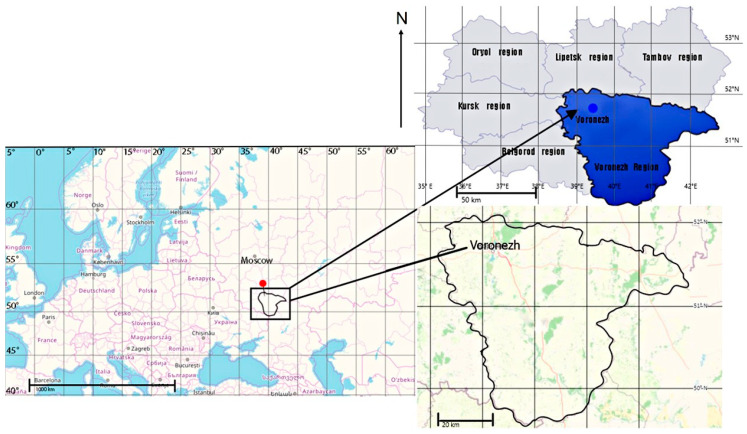
Scheme of the study area location.

Considering the diversity of the forest vegetation in the Central Forest Steppe, this study assessed forest stands growing in the forest steppe zone using the example of the Suburban Forestry of the East European Plain (using the example of the Voronezh Region). The objects of the research were selected based on similar phytocoenotic, ecological, and physical–geographical conditions of the Central Forest Stepperegion. This ensured that the sample was representative and that reliable results were obtained for the general population. The location of the study sites is shown in Figure 1.

**Figure 2:**
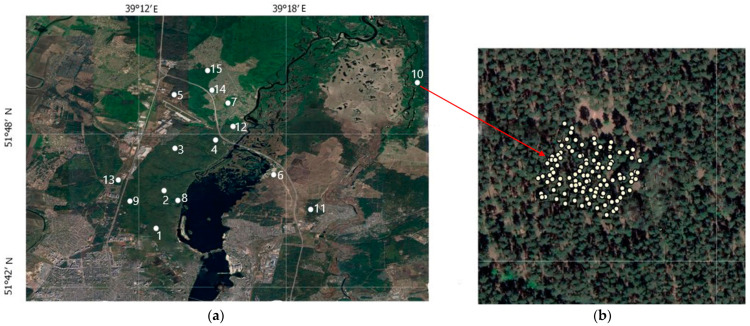
Location of trial plots on the territory of suburban forestry (numbers indicate the numberssampling plots): (**a**) sampling of the sites location; (**b**) placement of trees on SP 10.

The research program included the following steps:

1. Establishing sampling plots (Figure 2) in even-aged stands of different tree species composition and conducting the complete inventory on them with measurement of tree coordinates, species identification, trunk height and diameter, crown diameter, and tree state using ground-based inventory methods.
2. Performing aerial surveys using the RGB, hyperspectral, and Lidar imagery from the UAVs to determine similar silvicultural and taxation characteristics using remote sensing methods.
3. Generating training samples, designing computer vision models, and developing methods for determining the forest tree stand characteristics to calculate the phytomass of the forest stands using remote sensing methods.
4. Comparing and integrating forest and taxation parameters of the stands obtained during ground-based inventories and parameters of the forest areas obtained using the RGB, hyperspectral, and UAV-based Lidar surveys.
5. Application of the mathematical statistics and modelling methods to assess the accuracy of the results obtained, to verify the model validity, and to verify the developed algorithms.
6. Development of the methodology for remote sensing of aboveground phytomass of forest stands for subsequent calculation of carbon stock in the forest area.

Fifteen sample plots (50 × 50 m) were established in the coniferous, deciduous, and mixed forest stands located in different forest growth conditions with moderately moist (fresh) types according to P.S. Pogrebnyak and in the different forest types according to V.N. Sukachev classification [87]. We studied even-aged (conditionally even-aged) stands growing in the following forest growth conditions: A_2_ (fresh pine forest), B_2_ (fresh pine–oak forest), C_2_ (fresh pine forest with birch, aspen, and oak participation), C_2_D (fresh pine forest with oak participation), D_2_ (fresh oak forest). The age of the forest stands was 80–100 years. The tree stand characteristics of the studied stands are shown in Table 1.

### 2.2. Ground-Based Forest Inventory

The full count was performed in all sample plots to determine tree species and the coordinates of individual trees, trunk diameter, tree height, crown diameter, and tree health state. Undergrowth, shrub layer, and the degree of coarse woody debris were identified and recorded. 

The fieldwork was conducted utilizing modern geodetic and forest inventory equipment, including the SOUTH G2 GNSS receiver, Haglof Vertex Laser Geo altimeter. The methods employed were in accordance with the prevailing norms and legal frameworks in Russia [88,89,90].

Undergrowth and shrub layer were taken into account on the basis of the reforestation rules [91] and the forest management guidelines [92]. Undergrowth density degree (dense—over 8000 pcs. ha^−1^, medium—2–8000 pcs. ha^−1^, sparse—up to 2000 pcs. ha^−1^) and size categories (1—up to 0.5 m; 2—0.6–1.5 m; 3—over 1.5 m) were determined and species determination was performed. The shrub layer was estimated according to the following criteria: density degree (dense—more than 5000 pcs. ha^−1^, medium—2–5000 pcs. ha^−1^, sparse—less than 2000 pcs. ha^−1^), size (1—up to 1 m; 2—1–2 m; 3—more than 2 m), and species affiliation [90].

### 2.3. Estimation of the Aboveground Phytomass and Its Fractions

The estimation of aboveground phytomass stocks and its fractions (stem, branches, leaves) was conducted using allometric Equation (1) for each tree based on data on stem diameter at 1.3 m and tree height [93]:

ln P_i_ = a_0_ + a_1_ ln H + a_2_ ln DBH, (1)

where P_i_—phytomass stocks and its fractions (i; stem with bark, branches, needles/leaves), kg dry weight; H—tree height, m; DBH—stem diameter at 1.3 m, cm; a_0_, a_1_ and a_2_—species-specific coefficients of allometric equation.

To calculate the phytomass of pear (*Pyrus communis* L.), apple (*Malus sylvestris* L.), and mountain (*Sorbus aucuparia* L.) ash, the equation for bird cherry (*Prunus padus* L.) was used as the most closely related systematic category. The aboveground phytomass of undergrowth and shrubs with the DBH less than 8 cm was calculated for each species using their height according to allometric Equation (2) [94]:

P_ag_ = a × H^b^, (2)

where P_ag_—aboveground phytomass, kg dry weight; H—tree height, m; a and b—species-specific coefficients of allometric equation.

Statistical analysis was performed using STATISTICA-13 [95]. Pearson correlation (3) and ANOVA analysis (4), as well as mathematical modelling techniques, were used [96].

The study employed regression analysis methods [97]. Parameter estimates for linear models, as well as those reduced to linear form, were calculated using least squares, and nonlinear least squares for all other cases. Student’s *t*-test was used to test the null hypothesis of statistical significance for the estimated parameter values. Grouped data (by sample area) were analyzed using both linear and nonlinear mixed-effects models. The accuracy of linear mixed-effects models was assessed by conditional and marginal coefficients of determination (*R*^2^) [98] (see Equation(5)). Pearson *χ*^2^-test of agreement [99] was used to determine the consistency between the results obtained from the machine learning models and the actual distribution series obtained during fieldwork (see Equation(6)).

The following criteria were used to assess the quality of the models:

Correlation coefficient:

(3) $$R=\frac{\sum \left(x_{j}-x\right)\cdot \left(y_{j}-y\right)}{\sqrt{\sum \left(x_{j}-x\right)\cdot {\left(y_{j}-y\right)}^{2}}}$$

Reliability of differences:

(4) $$t=\frac{M_{1}-M_{2}}{\sqrt{m_{1}^{2}+\sqrt{m_{2}^{2}}}}$$

Coefficient of determination:

(5) $$R^{2}=1-\frac{\sum (y_{j}-y_{j})2}{\sum (y_{j}-y_{j})2}$$

Pearson agreement criterion:

(6) $$\chi^{2}=\sum \frac{(y_{j}-y_{j})2}{y_{j}}$$

where *M*—mean value, m—standard error; *x_i_*, *y_i_*—actual value; *ŷ_i_*—predicted value; *ŷ*—mean of actual values.

### 2.4. Flight and Survey Process 

The data array was generated using the UAV payload (using combined equipment—RGB, Lidar, hyperspectral camera, and GNSS receiver). The Lidar and hyperspectral survey were performedfrom an unmanned aerial vehicle (UAV), Luftera LQ-5 (take-off weight 9.5 kg); digital photography in visible spectrum was performed from an unmanned aerial vehicle (UAV), Luftera LQ-4 (take-off weight 5.2 kg). The spatial resolution of the orthophotomaps was 2–2.5 cm/pixel.

The flights were performed at two altitudes, 60–70 m and 120–130 m, with horizontal speeds not exceeding 3 m/s and 6 m/s, respectively, to ensure sufficient overlap at the shooting interval of the 1 frame per second and to minimize blur. Hyperspectral imaging was performed using a Cubert S185 hyperspectral camera. Each frame taken by this camera is a combination of a panchromatic image in JPG format with a resolution of 1000 × 1000 pixels and a geometrically coinciding hyperspectral data cube of 50 × 50 = 2500 spectra. The spectral range is 450–950 nm; the number of spectral channels is 125. 

### 2.5. Interpretation of the Tree Stand Characteristicsfrom High-Resolution Images

It should be noted that machine learning methods are not the primary focus of this paper. Moreover, the advances of machine learning methods have achieved such progress that they can be easily used in many practical problems. In this respect, the presented methods are well-known and universal, but require careful parameter tweaking for application for concrete forest plots.

For the neural network models, individual trees were labeled (tree species and its crown contour) on the small portion of each of the forest plots on the RGB geotiff-image. Data was prepared of the neural network classifier (bounding box rectangle were build)for each of the tree partitioning contours and data augmentation was applied for each of the images to improve robustness/generalizability. In particularsuch dataset was used to learn the model that was used to determine the tree species.

The neural network classifies the species of the individual trees. The condition and diameter of the crown were derived from this result. The computation of the hard-to-define taxation parameters was based on the identified relationships between the stand characteristics measured in the sample plots, the canopy digital model parameters, and the spectral characteristics of the aerial images. Training samples were generated from above mentioned labeled dataset and the YOLO neural network model was trained.

The characteristics of the forest stand were determined as follows:

- Tree canopy cover—by processing the RGB geotiffs;
- Tree height—by processing the Lidar imagery data;
- Precise tree location—by processing the Lidar imagery data;
- Tree species composition—from the RGB imagery using neural network models;
- Tree health state—with the hyperspectral data processing, based on the vegetation indices (NDVI, EVI, CVI);
- Crown diameter—using the RGB data processing and Lidar imagery;
- Stem diameter—by indirect indicators, based on the relationships identified using empirical relationships.

Traditional digital image processing techniques were used to separate trees from the background. The Lidar imagery uses geometric methods to interpolate the ground surface, namely by searching for the top of the forest canopy and then the height of the trees. Based on the Lidar imagery, the density of undergrowth and undergrowth in the stands was determined. 

The complex of the calculation parameters includes the simplest indices NDVI, EVI, and CVI (Equations (7)–(9)), which allowed us to assess the condition of the tree stands (to identify the presence of dead or weakened trees).

(7) $$NDVI=\frac{B_{\sim 0.8-0.9}-B_{\sim 0.63-0.75}}{B_{\sim 0.8-0.9}+B_{\sim 0.63-0.75}}$$

(8) $$EVI=2.5\times \frac{(NIR-RED)}{NIR+6RED-7.5BLUE+1}$$

(9) $$CVI=\frac{NIR}{GREEN}\times \frac{RED}{CREEN}$$

where B—intensity of spectral radiation in the corresponding bands (µm); *NIR*—reflection in the near infrared region of the spectrum; *RED*—reflection in the red region of the spectrum; *BLUE*—reflection in the blue region of the spectrum; *GREEN*—reflection in the green region of the spectrum.

### 2.6. Remote Estimation of Aboveground Phytomass and Carbon Stocks

The main objective of the remote estimation methodology for aboveground phytomass and carbon stocks was to significantly minimize ground surveys. The methodology is based on a sequence of steps that include the implementation of automated methods for the remote estimation of vegetation using UAVs, based on the application of machine learning models (use of artificial intelligence).

1. UAV-based determination using machine learning models:
  - Study area’s coordinates, terrain features, and delineation boundaries for forest inventory;
  - “Direct” silvicultural and stand characteristics, such as composition, height, crown diameter;
  - Condition and degree of debris, especially deadwood, in the stands.
2. Determination of “indirect” silvicultural stand characteristics such as age, stem diameter, canopy closure, and volume stock using reference materials and allometric equations, based on “direct” silvicultural and stand characteristics values.
3. Determination of the density of the undergrowth and shrub layer based on UAVs using the Lidar transect developed by the authors, as well as calculation of the phytomass of subordinate layers based on the generated tabular model.
4. Calculation of the carbon stock in the aboveground part of the forest stands (living and dead vegetation phytomass). Phytomass-to-carbon conversion factors were used according to IPCC guidelines [99]: 0.51 for coniferous tree species and 0.48 for broadleaved tree species.

## 3. Results

The methodological toolkit has been developed and a work algorithm has been defined that allows us to conduct remote assessment of the quantitative and qualitative forest characteristics with an accuracy that is comparable to that of traditional ground-based methods. The basis of the work algorithm is the integrated use of the Lidar, hyperspectral, and RGB survey materials using UAVs. This is necessary to obtain reliable information about the amount of aboveground phytomass and to calculate carbon reserves in the plantation by fraction. Information obtained online is extremely important for timely management decisions, for planning reforestation, and for environmental protection work.

### 3.1. Development of Technical and Methodological Tools for Determination of the Main Tree Stand Characteristics

#### 3.1.1. State of Tree and Shrub Vegetation

**Figure 3:**
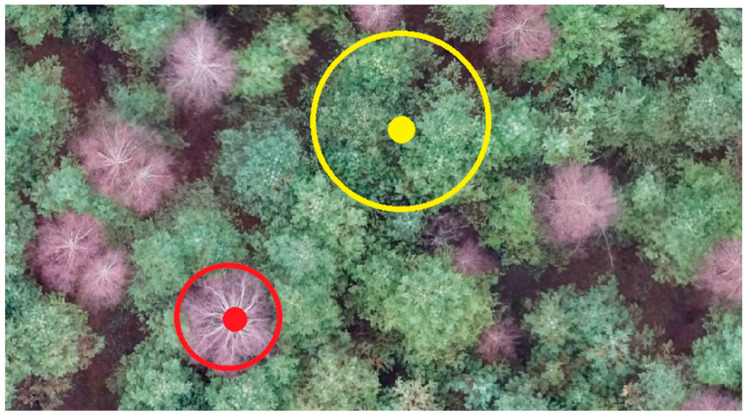
Selected areas for comparison of calculated indicators in the coniferous forest stands.

Determination of tree health category is based on vegetation indices calculation. The vegetation indices are constructed from hyperspectral data according to well-known formulas, based on the entire spectrum. Figure 3 shows selected areas in the forest area for the contrasting comparison and mapping of the proposed methodology. Dead trees are marked in red (NDVI-0.051), while fully viable trees are marked in yellow (NDVI-0.694).

The point quantitative data of the vegetation indices reflecting the qualitative characteristic of the vegetation condition in the selected areas are collected in Table 2. Higher index values correspond to denser and greener vegetation in the analyzed fragment. The plots with dead trees have lower index values (NDVI less than 0.15). Vegetation indices are characterized by the greatest accumulation of chlorophyll in the plants. Therefore, the higher this value, the better the sanitary condition of the planting and the phytocenoses formed by them.

**Figure 4:**
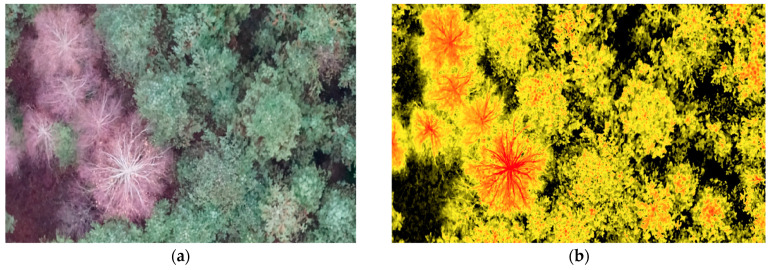
Fragment of images of trees with dead/lost foliage and healthy trees: (**a**) RGB photo; (**b**) superimposed NDVI calculation layer.

Figure 4 shows the forest plot with trees of different health categories. The calculated NDVI layer is superimposed on the original image, whereby the presence of dead and viable trees in the area can be identified: yellow (tree stand, understorey, shrub layer) reflects viable vegetation, black reflects dense ground cover, and red reflects the reduced NDVI value and allows for the identification of dead trees. 

#### 3.1.2. Determining Tree Coordinates and Measurement of Stem Height and Crown Diameter

The initial task of remote sensing data analysis (RGB complex, Lidar, and hyperspectral imagery) is to determine the location (coordinates) of trees in order to determine the main taxonomic characteristics of the tree stand.

**Figure 5:**
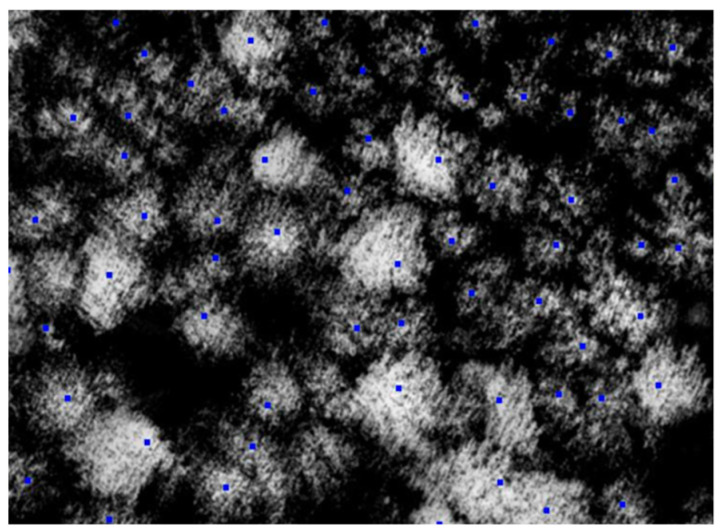
Maximums found on the elevation maps (marked in blue).

**Figure 6:**
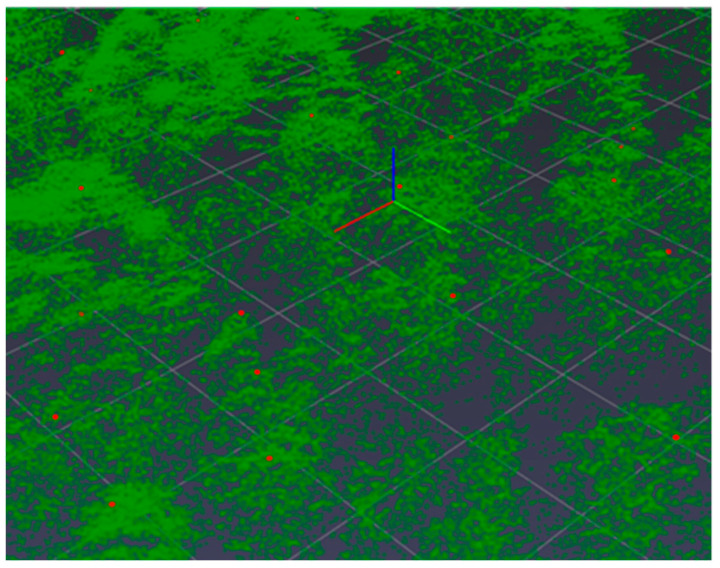
Formation of the height map (dots in green are trees, in red are their maxima).

The vegetation height matrices derived from the Lidar data are used for this purpose(Figure 5). The square window of fixed size is created in the center of each matrix cell; if the maximum of the window is reached at this point, the cell coordinates are declared to be tree coordinates, i.e., the tree coordinates coincide with the coordinates of their tops. Heights are then calculated at the derived points (Figure 6), and then the coordinates of the points are deduced from the coordinates of the las-file.

##### Tree Species Identification

**Figure 7:**
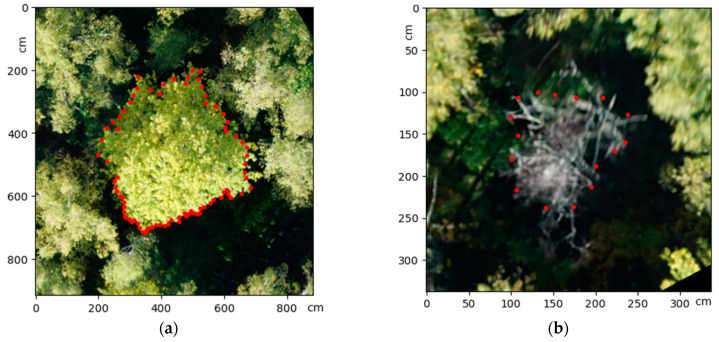
Outline from expert mark-up: (**a**) live tree; (**b**) dead tree(verification of crown contours is indicated by red dots).

Tree species identification was performed based on the computer vision process, consisting of expert partitioning of the tree species composition (Figure 7), training samples, and generating machine learning models based on convolutional neural networks on the YOLO architecture. Training an object-detection model such as the YOLOv5 requires a dataset containing object images and the coordinates of the bounding boxes of the objects themselves. 

The species composition of the forest stand was determined from RGB images using machine learning models. The algorithm for decoding species composition using artificial intelligence (based on neural networks) was conducted as follows:

1. Manual marking was completed—the operator carried out contour and analytical interpretation, as a result of which the species composition of the forest stand was determined.
2. Integration of manual marking data and RGB images.
3. Formation of training samples using neural network analysis.
4. Testing and calibration of machine learning models created based on neural networks.
5. Hyperspectral data were used to clarify the species identity and correct the breed composition.

##### Determination of Crown Diameter

**Figure 8:**
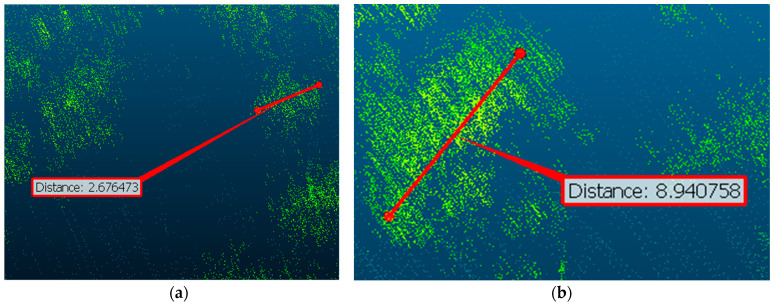
Measurement of crown diameters (meters) from the Lidar data: (**a**) tree crown diameter on SP 13; (**b**) tree crown diameter on SP 3.

The tree crown diameter decoding process was based on the processing of point clouds obtained during the Lidar imagery processing. Knowing the coordinates (centers) of the trees, it is possible to determine both the heights and the crown diameters of individual trees. Pre-processing is performed manually in specialized point cloud software (e.g., QGIS, https://qgis.org/ru/site/ accessed on 8 May 2024) (Figure 8).

Following training of the AI-based on the neural network method, crown diameters were determined automatically. 

##### Determination of Tree Height and Height of Crown Base

**Figure 9:**
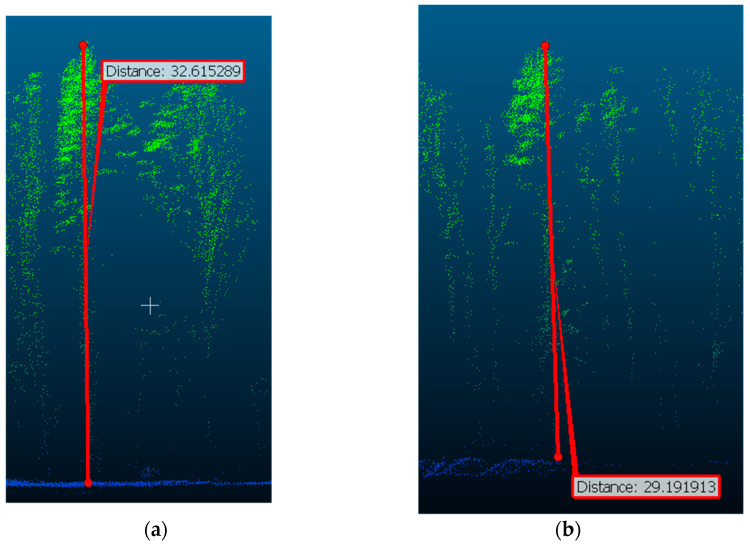
Measurement of tree height (meters) from the Lidar data: (**a**) maximum tree height on SP 8; (**b**) maximum tree height on SP 10.

The process of determining tree heights was based on working with the Lidar point clouds. Each point cloud file contains information about the coordinates of the points, which allows us to determine the ground surface and the forest canopy; by subtracting one from the other, one candeterminethe stand height in meters (Figure 9).

Automated tree height estimation involves calculating ground and vegetation surfaces, and then estimation of heights is performed by subtraction of the ground surface coordinate from the vegetation surface. 

#### 3.1.3. Shrub Layer and Understory Phytomass Assessment

The shrub layer and the understory in the stand are not always fully accounted for in carbon balance estimations [65,100,101,102,103]. However, the density and height of undergrowth can have a significant impact on the carbon content of phytomass. To improve the accuracy of determining the forest stand phytomass, the scale for accounting undergrowth and undergrowth by remote sensing methods using hyperspectral and the Lidar survey data was developed.

**Figure 10:**
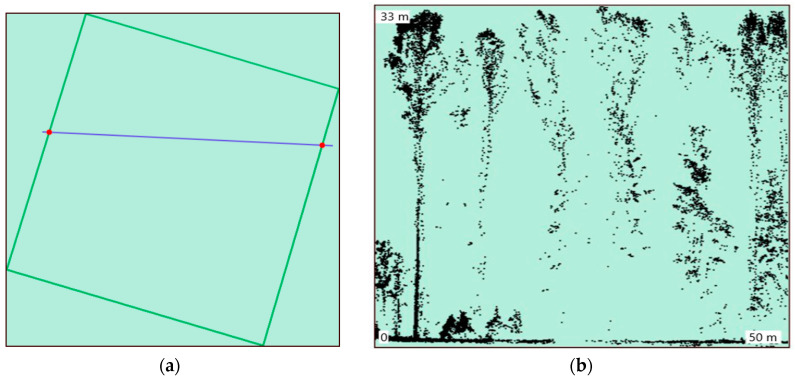
Displaying of the Lidar data in the web interface: (**a**) general view and location of the Lidar section in the study area; (**b**) three-dimensional structure of the forest stand displayed as the Lidar transect.

To calculate the amount of undergrowth using remote methods, a Lidar section was created (Figure 10), covering an area of 50 × 1 m. Based on the obtained value from Table 3 and Table 4, the phytomass was calculated.

The scale was compiled on the basis of the experimental materials (field samples obtained both in this study and in earlier studies), with the application of mathematical modelling methods for interpolation and approximation of the results. The phytomass was calculated considering categories of density and height (see Table 3 and Table 4).

The Lidar transect was performed using the developed tools with the web interface shown in Figure 10a. At least three Lidar transects should be made in the studied area (Figure 10b) and then the average number of shrubs and understory should be determined.

**Figure 11:**
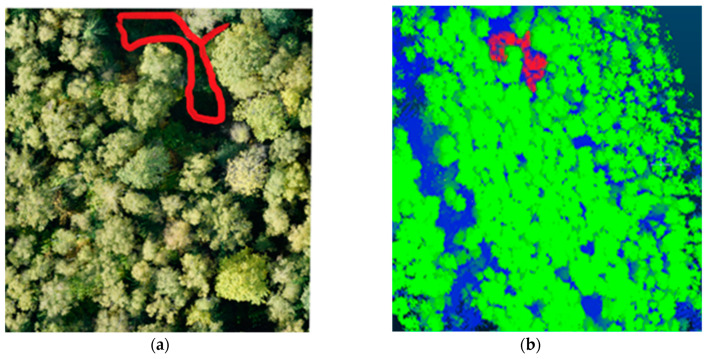
Combining the RGB (**a**) and Lidar (**b**) images by common attributes.

The scientific novelty of working with hyperspectral cubes is their processing algorithm. Clustering of spectra for each image is performed; here, 2500 spectra are grouped into several clusters with the k-means clustering algorithm. The resulting median spectra are compared with the spectra of reflected light from the plants, on the basis of which they are combined into clusters. The spectra belonging to the clusters that clearly do not correspond to the plant spectra are then excluded from further consideration, while the remaining spectra are involved in computer vison methods. In partitioning mode, training samples are generated from them. The example of the complex data processing is shown below (Figure 11). 

**Figure 12:**
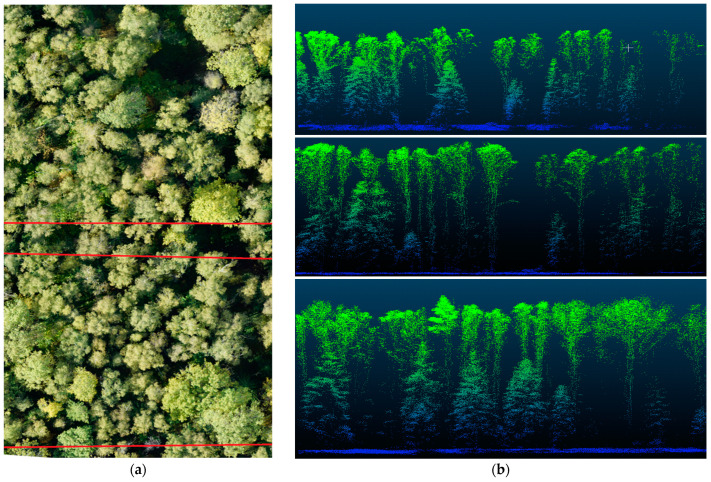
Analyzed cross-section lines (**a**) and their mapping after processing the Lidar data (**b**) in stand studies.

The hyperspectral and Lidar devices provide separate categories of data to describe forests at individual tree level. The hyperspectral imagery contains meaningful plant reflectance characteristics or spectral features, while Lidar data enables the analysis of a canopy’s structural properties. Combining these two data sources improves the quality of the forest mapping (Figure 12).

This requires different data collection methods, appropriate processing algorithms, and classification and/or regression techniques. Remote sensing method integration aims to generate integrated information based on data with different spatial and spectral resolutions. These integrated data are more reliable and accurate than individual sources of information. Consequently, their integration improves the quality of the work and provides increased accuracy in calculating phytomass stock and estimating the carbon storage of the forest stands.

### 3.2. Determination of Correlations between Stand Characteristics in Order to Indirectly Identify the Deciphering Characteristics of the Forest Stand for Analytical Interpretation

Tree stem diameter was determined by indirect methods based on the empirical data and developed regression equations. Crown diameter and tree height have a diagnostic function and are in close correlation with the stem diameter at breast height. This makes it possible to develop regression models to estimate various characteristics of individual trees, which can then be used in aerial monitoring. 

**Figure 13:**
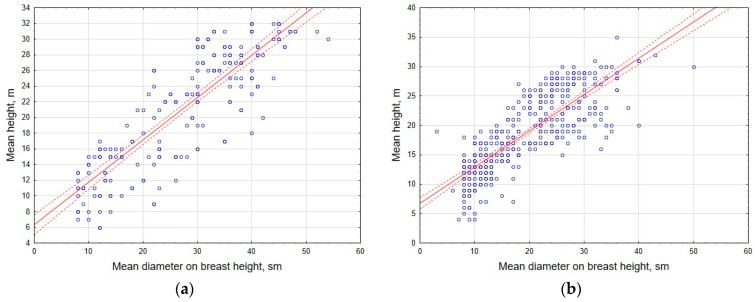
Correlation of stem diameter and tree height in coniferous (**a**) and deciduous (**b**) tree stands.

**Figure 14:**
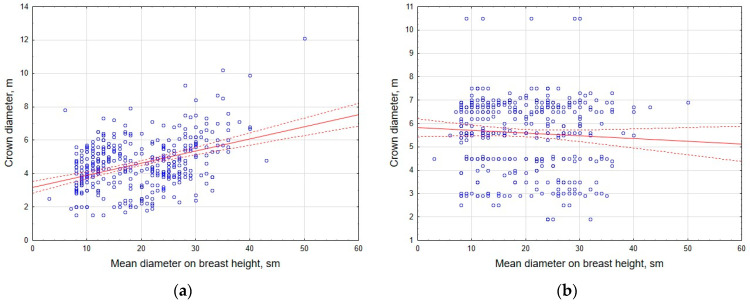
Correlation of crown diameter and stem diameter in coniferous (**a**) and deciduous (**b**) tree stands.

Preliminary correlation analysis of the data has shown that, for individual tree species, there is a fairly close relationship between stem diameter at 1.3 m height and crown diameter (R = 0.56 and 0.67 in coniferous and deciduous plantations, respectively) or tree height (R = 0.71 and 0.68); the strength of the relationship with the height parameter was generally higher than the corresponding relationship with crown diameter (Figure 13 and Figure 14).

Tree height and crown diameter are essential for remote forest monitoring since they can be accurately determined from the RGB and Lidar imagery. Based on the results of the multivariate regression analysis, models were developed to determine tree stem diameter at 1.3 m using these most informative morphometric indicators of trees (see Equation(10)). The results of the calculation of the equation coefficients are summarized in Table 5.

Y = a_0_ + a_1_ H + a_2_ D_cr_ (10)

where Y—stem diameter at breast height 1.3 m (cm); H—tree height, m; D_cr_—average crown diameter, m; a_0_, a_1_, and a_2_—regression coefficients.

The calculated coefficient of determination (R^2^) in the considered options exceeded 0.84 and confirmed the agreement of the linear regression models with the experimental data. The exception is oak (R^2^< 0.5), which requires an adjustment to the model used.

**Figure 15:**
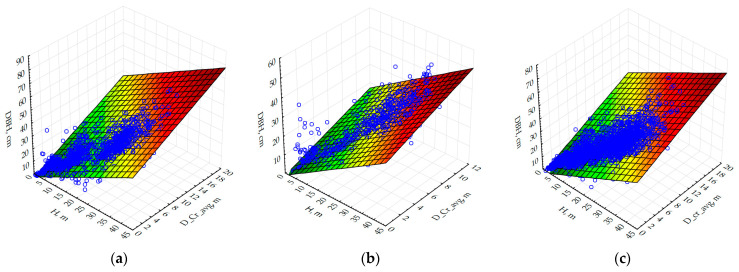
Relationship between stem diameter (DBH, cm) and mean crown diameter (D_cr_avg, m) and tree height (H, m) for main tree species in the Central Forest Steppe of the East European Plain for different tree species: (**a**) Scots pine (*Pinus sylvestris* L.); (**b**) birch (*Betula pendula* L.); (**c**) aspen (*Populus tremula* L.).

The results of the regression analysis improve the explanatory capacity of the models by 30–40% when several of the most significant morphometric indicators are used for prediction. Therefore, preference should be given to two- and multifactor models. Calculations based on the developed allometric models provide the acceptable error in determining stem diameter of almost all tree species (except for oak)—for aspen, pine, and birch, the equations provide the most accurate estimates of the considered taxation characteristics (Figure 15) and therefore could be applied in the analysis of the Lidar survey results.

### 3.3. Validation of the Proposed Phytomass Estimation Toolkit

The next stage of the study comprised testing the toolkit developed to determine the phytomass of the forest stands. The comparison was made between the results obtained by interpretation of imagery and the results of the ground-based inventory of trees on the sample plots. 

The estimation of the phytomass by remote sensing was carried out using data on species composition and stand health (based on the RGB and hyperspectral imagery), stem diameter (derived from the allometric equations), and stand height (derived from the Lidar imagery). Average data from Table 4 and Table 5 were used to calculate understory and shrub phytomass. Additionally, dead wood was taken into account, based on the RGB and hyperspectral surveys. 

The detailed data on the amount and fractional structure of phytomass obtained by ground-based inventory of sampling plots are shown in Table 6.

An analysis of Table 6 suggests that the maximum aboveground phytomass stocks in the forests of the Voronezh Region were in mixed oak–pine stands, reaching 303.0–340.3 t ha^−1^ (SP 1 and SP 8). Significant stocks of the aboveground phytomass (up to 309.4 t ha^−1^) were also found in the birch forest with ash and linden. The lowest values of the phytomass were observed predominantly in the coniferous stands (dominated by Scots pine)—up to 153.2 t ha^−1^ (SP 10, SP 11). 

**Figure 16:**
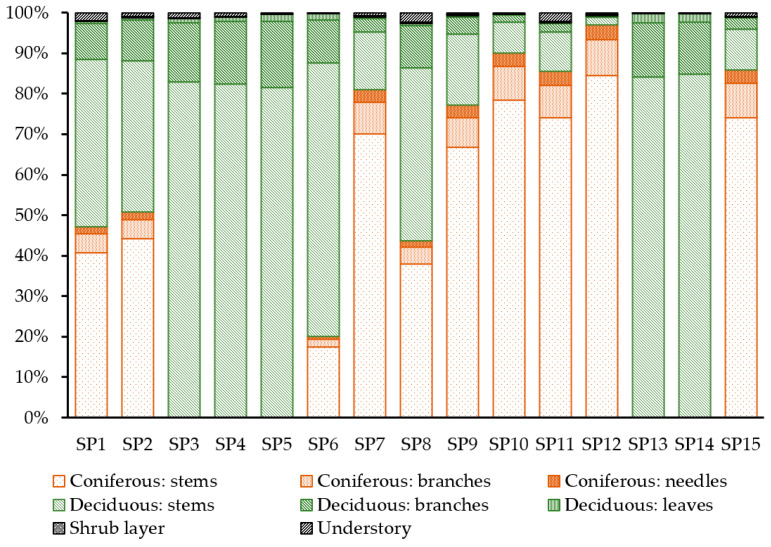
Fractional composition of the phytomass on the sample plots. Sample plot numbers are marked on the horizontal axis.

Understory and shrub layer together contribute about 1–2% of total phytomass. The lowest contribution of the lower forest layers to the total forest stand phytomass was observed in mixed birch–pine forest stands—0.2% (SP 6, SP 13)—and the highest proportion of the total phytomass was in subcanopy layers of mixed oak–pine forest stands—2.6% (SP 8) (Figure 16).

Generally, the fraction in tall (over 2 m in height) undergrowth tends to be higher than in the comparable category of undergrowth. The undergrowth fraction varies over the wide range of two orders of magnitude, from 75 to 8044 kg ha^−1^. Contribution of the undergrowth to the total phytomass on the investigated sample plots was not significant and did not exceed 0.5%. Its stocks vary from 142 kg ha^−1^ in mixed birch–pine forests to 954 kg ha^−1^ in mixed stands with Scots pine predominance.

Stem wood accounts for at least 81% of the total phytomass of the stand. The analysis of the ratio of woody phytomass fractions clearly shows the pattern of increasing contribution of branches in mixed forests with the predominance of deciduous species, and deciduous forests—up to 15–16%—while the proportion of branches in coniferous forests accounts for less than 9–10% of total phytomass. 

**Figure 17:**
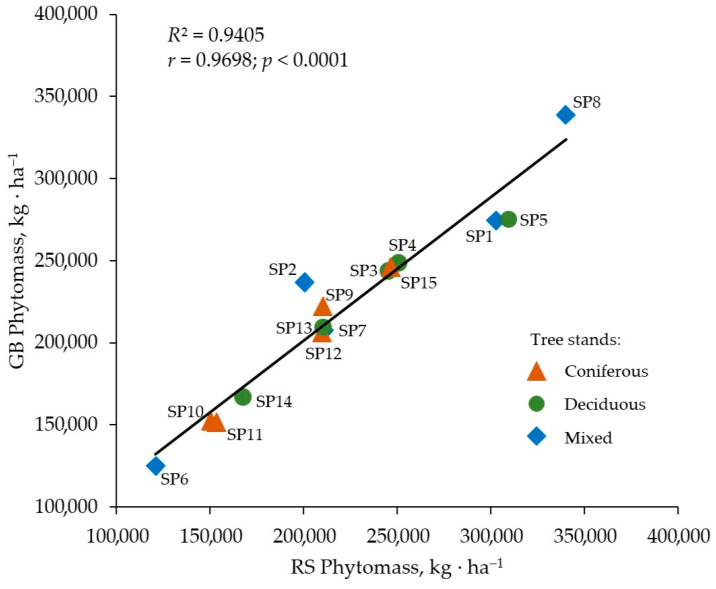
Total phytomass of different tree stand types on the sample plots (SP) determined by ground-based (GB) and remote sensing (RS) methods.

The statistical processing of the results was performed using the ANOVA (Table 7) to determine the significance of differences between the average values of the tree stand characteristics and the total amount of phytomass in stands, determined by ground-based and remote sensing methods (Figure 17).

Significant differences (at the significance level of *p* < 0.05) between the average values of the total phytomass from the ground-based and remote sensing methods were found in 33% of the cases. However, the maximum difference (observed at SP 5) in the approximation of the data did not exceed 15%. In 66% of the cases, the average phytomass values determined by the different methods did not differ significantly from each other. This demonstrates the high accuracy of the information obtained by remote sensing, using the proposed technical–methodological approach. 

Remote sensing performed on the basis of the proposed methodology is comparable in accuracy to ground-based methods for determining the carbon stock in aboveground phytomass. This is achieved by taking into account undergrowthand dead wood.

The similarity of phytomass values obtained by the ground-based and the remote sensing methods was statistically confirmed: Pearson criterion *χ*^2^ (0.05; 5) = 2.79; *P*(*χ*^2^) = 0.68 > 0.05 was found to causeslight differences in the results of calculating phytomass by different methods. Consequently, we can reliably use the distance-based phytomass estimation technique to calculate the carbon storage of forest stands. 

### 3.4. Calculation of Carbon Stock from Phytomass Measurements

The carbon stock calculations for the aboveground phytomass of the forest stands in the sample plots are summarized in Table 8.

The total carbon stocks in the forest stands varied from 58.6 t ha^−1^ in birch-dominated stands (SP6), where minimal stem wood stocks were observed, to 167.8 t ha^−1^ in mixed pine-dominated stands (SP8). The shrub layer and understory contributed no more than 3.8 t C ha to the total carbon stocks. In most cases, the carbon stocks in their aboveground phytomass did not exceed 1.0 t ha^−1^. 

## 4. Discussion

The hyperspectral and Lidar data complement each other. The synergy between the technologies used can be referred to as 3D-imaging spectroscopy, which has been explored in the works of several authors [104,105]. The proposed methodology is primarily based on the use of the RGB and Lidar sensors estimating as much of the forest stand phytomass as possible and allows consideration of all carbon pools for the rapid and reliable calculation of the carbon storage [106,107,108].

Similar methods for remotely estimating stands and determining the accumulation of forest phytomass using the Lidar system were used for the tree species *Quercus semicarpifolia* (Sm.) and *Pinus roxburghii* (Sarg), when calculating the carbon stock in the subtropical and temperate forests of India [108,109,110]. The proposed method was based on the assessment of ground phytomass and carbon only for the tree layer, which is not appropriate and objective when assessing forest phytocoenosis as the whole. Please note that the machine learning models used are developed on a regional basis.

In monitoring the ecological state of vegetation and carbon reserves, great importance belongs to the qualitative and quantitative characteristics of the productivity of aboveground phytomass of individual plants and phytocoenoses. Moreover, in contrast to the traditionally implemented assessment of the carbon pool in the aboveground phytomass of tree stands [33,34,64], we have studied the parameters of the accumulation of aboveground phytomass and carbon in other tiers of the forest phytocoenoses. This also indicates the feasibility and objectivity of the assessment.

In addition to the required technical and software tools, adequate methodological apparatus is required. In our case, a comprehensive technical and methodological approach was used. We believe that the assessment required data from our ground-based monitoring on the morphometric characteristics of plants and their condition. This was confirmed by a correlation analysis. Positive correlations between the morphometric parameters of the woody plants were obtained. In particular, the parameters of tree height and crown diameter are important for remote environmental monitoring of the forest phytocoenoses. They can be determined by ground-based methods and remotely (from RGB images and through Lidar surveys). Multivariate regression analysis makes it possible to develop models for determining the diameter of the tree trunk, using morphometric parameters. The values of the species-specific coefficients for the number of the tree species are given. Priority should be given to two- and multifactor models when predicting the necessary morphometric parameters of ecologically and economically valuable plants. The validation of the proposed set of tools is disclosed and explanations are given for estimating phytomass using the example of the forest phytocoenoses.

The data obtained from ground-based monitoring and remote monitoring were compared differentially for the tree stands, undergrowth, shrub layer, and in general (Table 6). The major advantage of the methodology proposed for remote monitoring and control of the carbon storage compared to existing analogues is the minimization of the amount of groundwork and, consequently, the reduction inlabor costs without loss of information quality. Reliable information on the phytomass volumes will allow for operational control of forest carbon storage, which is essential for timely management decisionmaking. The proposed methodology is useful for analyzing the possibilities of sequestration of carbon and other elements in plant phytomass and soils in forested, agricultural, and populated areas. Of great importance is the application of the described methodology for studying the ecosystem-related, ecological–protective, and phytomeliorative properties of tree stands and other vegetation elements in relation to agrophytocoenoses. 

Wu et al. [111], Reinmann et al. [112], Junfang et al. [113], and thenumber of other researchers [114,115,116] believe that the quantification of the aboveground forest phytomass and carbon cycle studies in general is a highly relevant and current issue, which will provide the theoretical basis for the study of the carbon cycle and global climate change. Obtaining information on the forest ecosystems and carbon storage will make it possible to identify the inverse relationship between the processes occurring in forests and climate change. It also seems promising to analyze the state of tree plantations and forests, the accumulation of their phytomass to consider the balance of the carbon and nutrient cycles, to assess degradation processes due to environmental and climatic transformations in the environment and in the forest natural ecosystems and cultural ecosystems [117,118,119]. Data on the formation of the phytomass of the forests and plantations can be used as the basis for environmental and climate–economic management of natural and cultural ecosystems. This is consistent with the results of other authors [120,121,122,123]. This seems possible in geographically, ecologically, and economically diverse areas. The presented methodology for integrated carbon monitoring allows us to identify the ecological state, the limits of sustainability, and the resource base of the corresponding natural phytocoenoses and agrophytocoenoses. The proposed universal methodology can be of decisive importance in the selection of plant species and varieties for afforestation, landscaping, horticulture, protection, and restoration of soil ecosystems, and the protection of and increase in the resource attractiveness of landscapes on the East European Plain and other territories.

## 5. Conclusions

The use of UAVs for the remote sensing of forest ecosystems in the Eastern European Plain is the issue that is becoming more and more important. Estimating the amount of carbon stored in forests is the critical undertaking. At present, there is no single system that integrates the collection, storage, and analysis of data on forest ecosystem health and structure, leading to significant uncertainties in current biomass assessment methods. It is crucial to enhance existing methods and develop new approaches for estimating forest phytomass and calculating carbon stocks.

The estimation of the carbon storage of the forests is based on the calculation of the phytomass of the stand and other components of the forest ecosystem. Meanwhile, woody phytomassis based on the application of allometric equations or conversion factors, defined as the ratio of total stand phytomass (including aboveground, belowground, and understory) to the stemwood stock. The conversion factors are differentiated according to the age of the stand and other taxonomic indicators obtained by ground-based forest inventory.

In the study area, on average, up to 81% of the total volume of formed phytomass is stem wood. To complete the assessment, the contribution of other layers of forest vegetation was also established. The undergrowth and shrub layer are responsible for the formation of aboveground phytomass in the volume of up to 2% of the total volume of aboveground phytomass of forest phytocenoses. Phytocenoses of the oak, oak–pine, pine, and birch forests are natural ecological frameworks for the ecosystems they form and for the ecosystems adjacent to them. The total carbon reserves in the aboveground phytomass of phytocenoses varied, depending on their composition. The total carbon reserve reached its maximum value (163.6 t ha^−1^) in plantations of mixed species composition with the predominance of the pine.

To integrate and approximate data obtained during ground surveys and data obtained during remote monitoring based on the UAVs, technical and methodological tools have been developed. This toolkit allows you to analyze and decipher information. The technical and methodological tools used in forest sensing using the UAVs are based on machine learning technology using the neural network method. The proposed integrated approach makes it possible to determine taxation indicators of plantings and volumes of aboveground phytomass in the automated mode (or with elements of automation). To achieve automation of the process, data of three categories (RGB, Lidar, hyperspectrum) were collected and artificial intelligence methods based on the ultra-precise neural network were applied.

The remote determination of the main forest stands’ taxation indices and, accordingly, the volume of the phytomass of the key carbon pools will also make it possible to remotely determine the carbon storage of forest ecosystems. This will allow the remote monitoring of carbon storage over large areas.

The similarity of phytomass values obtained by ground-based and remote sensing methods was statistically confirmed: Pearson criterion *χ*^2^ (0.05; 5) = 2.79. Consequently, we can reliably use the distance-based phytomass estimation technique to calculate the carbon storage of forest stands.

The use of the UAVs with appropriate hardware will make it possible to obtain the forest characteristics necessary for the determination of the carbon storage as the need arises: to determine baseline characteristics at the start of forest climate projects, when monitoring the dynamics of carbon storage, and so on. It is also possible to determine the characteristics of forests after selective logging and after fires of different intensities. Of great importance is the possibility of using the scientific idea of the work and the described methodology to assess and predict the condition of the trees and shrubs as ecological frameworks and complex phytomeliorants on lands of different target categories. The methodological approach we propose is easily compatible with current technologies in the field of conservation, protection, and reproduction of forests in the Central Forest Steppe Region in the East European Plain. This allows the approach to be successfully integrated into the general system of forest monitoring and the assessment of stocks of wood and other plant resources, and into environmental and economic management in different types of cultural and natural ecosystems and in different landscapes.

## Figures and Tables

**Table 1:** Species composition, tree stand characteristics, and forest growth conditions of the sample plots.

No of Sample Plot	Location	Composition of Tree Species, %	Age, Years	DBH, cm	H, m	f	Volume Stock, m^3^ ha^−1^	FGC
1	Quarter 44; site 26	70% Scots pine 30% English oak	90	35	27	0.6	266	C_2_
2	Quarter 51; site 44	80% Scots pine 20% English oak	90	31	28	0.6	220	C_2_
3	Quarter 27; site 14	90% English oak 10% Linden	90	30	26	0.6	250	C_2_D
4	Quarter 11; site 25	80% English oak 20% English oak	90	28	24	0.6	210	D_2_
5	Quarter 6; site 11	100% European birch	85	40	28	0.6	210	C_2_D
6	Quarter 110; site 13	90% European birch 10% Scots pine	75	22	23	0.7	190	A_2_
7	Quarter 9; site 28	80% Scots pine 20% European birch	90	30	26	0.7	310	C_2_
8	Quarter 46; site 10	80% Scots pine 20% English oak	90	32	28	0.8	350	B_2_
9	Quarter 48; site 44	80% Scots pine 20% Scots pine	90	30	24	0.8	330	A_2_
10	Quarter 6; site 3	100% Scots pine	90	28	25	0.7	320	A_2_
11	Quarter 76; site 21	100% Scots pine	90	30	26	0.6	290	A_2_
12	Quarter 78; site 7	100% Scots pine	90	31	28	0.7	350	B_2_
13	Quarter 48; site 79	100% Aspen	95	32	26	0.7	290	C_2_D
14	Quarter 8; site 10	100% Aspen	100	30	26	0.6	230	C_2_D
15	Quarter 48; site 17	100% Scots pine	95	32	28	0.7	350	B_2_

Note: H—mean height; DBH—mean diameter on breast height; f—density of tree placement in the PRP (corresponds to the canopy density); FGC—forest growth conditions.

**Table 2:** Calculation of some indices for selected areas.

Vegetation Index	Indicator Value in the Red Area	Indicator Value in the Yellow Area
NDVI	0.0515	0.6944
CVI	1.1382	2.9996

**Table 3:** Reference values of the shrub phytomass in the stands growing in the fresh forest growth conditions, kg ha^−1^ dry weight (range of variation/mean value).

Criteria	Density Degree, pcs. ha^−1^								
	Dense (More than 5000)			Medium (2–5000)			Sparce (Less than 2000)		
Number, pcs. transect^−1^	>25			11–25			1–10		
Average height, m	>2.0	1.1–2.0	<1.0	>2.0	1.1–2.0	<1.0	>2.0	1.1–2.0	<1.0
Shrub phytomass in coniferous stands	600–4900 2600	125–1900 900	50–400 180	550–1800 1050	50–650 300	5–140 90	10–1100 400	5–250 100	1–50 20
Shrub phytomass in deciduous stands	1500–5200 3000	150–2650 1100	60–760 200	600–2500 1400	60–700 500	5–150 100	10–1500 650	5–550 200	1–50 25
Shrub phytomass in mixed stands	1250–5200 2950	150–2600 1050	50–600 190	600–1920 1260	50–650 400	5–150 100	10–1325 500	5–470 180	1–50 20

**Table 4:** Reference values of the understory phytomass in the stands growing in the fresh forest growth conditions, kg ha^−1^ dry weight (range of variation/mean value).

Criteria	Density Degree, pcs. ha^−1^								
	Dense (More than 8000)			Medium (2–8000)			Sparce (Less than 2000)		
Number, pcs. transect^−1^	> 40			11–39			1–10		
Average height, m	>1.5	0.6–1.5	<0.5	>1.5	0.6–1.5	<0.5	>1.5	0.6–1.5	<0.5
Understory phytomass in coniferous stands	5000–22,000 7500	500–9900 1900	100–900 450	1100–13,500 2500	90–6100 750	20–300 150	10–3950 550	5–1150 150	1–50 25
Understory phytomass in deciduous stands	3500–24,000 6000	380–5500 2000	100–800 400	800–18,000 2700	60–3500 700	20–250 130	10–5500 750	5–850 170	1–40 25
Understory phytomass in mixed stands	3200–16,000 5900	520–6200 1800	100–750 400	800–8400 2600	50–3200 700	30–250 130	10–3600 650	5–800 160	1–40 20

**Table 5:** Linear regression equation constants (a_0_–a_2_) for calculating stem diameter of different tree species.

Tree Species	Regression Coefficients			R	R^2^	SE
	a_0_	a_1_	a_2_			
Scots pine (*Pinus sylvestris* L.)	–2.7626 ± 0.1490	0.8739 ± 0.0123	2.2386 ± 0.0653	0.955	0.913	4.055
Oak (*Quercus robur* L.)	2.4860 ± 1.7332	1.0969 ± 0.0781	0.4070 ± 0.2163	0.634	0.402	9.039
Birch (*Betula pendula* L.)	–3.8368 ± 0.3637	0.6860 ± 0.0247	2.2120 ± 0.1015	0.920	0.846	4.223
Aspen (*Populus tremula* L.)	–3.6842 ± 0.4640	0.8152 ± 0.0626	1.9195 ± 0.2784	0.965	0.932	3.820

Species-specific coefficients are represented as a_n_ ± 95% confidence interval.

**Table 6:** Phytomass stocks of the forest stands (age of 90–100 years) on the sample plots (SP) in the fresh forest growth conditions of the Central Forest Steppe of the East European Plain (kg ha^−1^ dry weight).

No of SP	Tree Stand, Including							Shrub Layer	Understory	Total Phytomass
	Stems		Branches		Needles/Leaves		Total			
	M ± SE	Total	M ± SE	Total	M ± SE	Total				
1	494 ± 21	248,800	80 ± 6	40,368	15 ± 0.8	7342	296,510	418	6078	303,006
2	404 ± 23	163,127	73 ± 7	29,467	13 ± 0.9	5091	197,686	549	1954	200,189
3	513 ± 29	203,294	90 ± 6	35,634	7 ± 0.3	2644	241,572	461	3025	245,059
4	493 ± 29	206,855	92 ± 6	38,447	7 ± 0.4	2757	248,060	622	2121	250,804
5	644 ± 31	252,281	129 ± 8	50,576	14 ± 0.8	5467	308,324	168	926	309,418
6	224 ± 13	102,361	33 ± 2	15,031	7 ± 0.4	2974	120,366	142	75	120,583
7	345 ± 21	177,882	46 ± 4	23,482	14 ± 0.9	7309	208,672	954	1480	211,107
8	483 ± 28	274,535	86 ± 9	49,100	14 ± 0.8	7874	331,508	764	8044	340,317
9	288 ± 12	177,569	39 ± 3	23,832	12 ± 0.4	7400	208,801	562	998	210,361
10	288 ± 15	128,924	33 ± 2	15,003	12 ± 0.6	5526	149,452	210	324	149,987
11	315 ± 14	128,454	38 ± 2	15,388	14 ± 0.6	5584	149,426	510	3259	153,194
12	351 ± 15	180,927	37 ± 2	19,339	15 ± 0.7	7710	207,976	665	1018	209,659
13	378 ± 8	177,015	60 ± 2	28,023	10 ± 0.3	4781	209,819	347	142	210,308
14	362 ± 17	141,827	55 ± 4	21,487	9 ± 0.4	3352	166,667	281	246	167,193
15	414 ± 20	207,249	55 ± 4	27,474	18 ± 1.1	8774	243,497	381	2478	246,356

M—mean value of the sign; SE—standard error.

**Table 7:** Significance of differences between the mean values of phytomass in the forest stands (t_a-b_) determined by ground-based (a) and remote sensing (b) methods.

Type of Tree Stands	Sample Plots (Differences in Phytomass, a–b)															t_0.05_
	**1**	2	3	4	5	6	7	8	9	10	11	12	13	14	15	
Coniferous									−1.6 *	−1.7 *	0.8 *	2.3			0.2 *	1.96
Deciduous			1.7 *	1.8 *	5.5								0.5 *	1.4 *		1.96
Mixed	5.2	−3.1				−2.6	1.8 *	0.6 *								1.96

* Insignificant differences between the means.

**Table 8:** Aboveground carbon stock of the forest stands (age of 90–100 years) on the sample plots (SP) in the fresh forest growth conditions of the Central Forest Steppe of the East European Plain (kg ha^−1^).

No of SP	Tree Stand, Including				Shrub Layer	Understory	Total Carbon Stock
	Stems	Branches	Needles/Leaves	Total			
1	123,128	19,794	3690	146,612	200	2918	149,730
2	80,952	14,427	2559	97,937	263	938	99,139
3	97,581	17,104	1269	115,954	221	1452	117,628
4	99,291	18,455	1323	119,069	299	1018	120,386
5	121,095	24,276	2624	147,995	81	444	148,520
6	49,766	7282	1457	58,504	68	36	58,608
7	89,823	11,767	3708	105,297	458	711	106,466
8	135,648	23,991	3947	163,586	367	3861	167,814
9	89,450	11,896	3748	105,093	270	483	105,846
10	65,411	7574	2808	75,793	101	156	76,049
11	65,061	7754	2836	75,652	245	1564	77,460
12	92,159	9843	3928	105,931	319	490	106,740
13	84,967	13,451	2295	100,713	166	68	100,948
14	68,077	10,314	1609	80,000	135	118	80,253
15	104,951	13,814	4460	123,225	183	1192	124,600

## Data Availability

The data presented in this study are available on request from the corresponding author. The data are not publicly available due to privacy restrictions.
